# A strengths based method for homeless youth: Effectiveness and fidelity of Houvast

**DOI:** 10.1186/1471-2458-13-359

**Published:** 2013-04-18

**Authors:** Manon AM Krabbenborg, Sandra N Boersma, Judith RLM Wolf

**Affiliations:** 1 Radboud University Nijmegen Medical Centre, Department of Primary and Community Care, Netherlands Centre for Social Care Research, PO Box 9101, Nijmegen, 6500 HB, The Netherlands

**Keywords:** Homeless youth, Strengths based method, Quality of life, Mental health, Substance use, Fidelity, Quasi-experimental design

## Abstract

**Background:**

While homelessness among youth is a serious problem, there is little information about evidence-based interventions for homeless youth. In cooperation with professionals and youths, Wolf (2012) developed Houvast (Dutch for ‘grip’): a strengths based method grounded in scientific and practice evidence. The main aim of Houvast is to improve the quality of life of homeless youths by focusing on their strengths, thus stimulating their capacity for autonomy and self-reliance.

**Method/Design:**

The effectiveness and fidelity of Houvast will be tested in ten Dutch services for homeless youth which are randomly allocated to an intervention group (*n* = 5), or a control group which provides care as usual (*n* = 5). Measurements of both objective and subjective quality of life and secondary outcomes (mental and physical health, substance use, coping, resilience, psychological needs, care needs, working relationship with the professional and attainment of personal goals) will be conducted among homeless youths (*n* = 251). Youths in both groups will be interviewed by means of a structured interview at baseline, at time of ending care or after having received care for six months (T1) and at nine months after baseline (T2). Model fidelity will be tested around T1.

**Discussion:**

This study is unique as it includes a large number of homeless youths who are followed for a period of nine months, and because it focuses on a strengths based approach. If the Houvast method proves to be effective in improving quality of life it will be the first evidence-based intervention for homeless youth.

**Trail registration:**

Netherlands Trail Register (NTR):NTR3254

## Background

Homelessness among youth is a serious problem in many countries. Although, some researchers estimate that as many as 50000 homeless adolescents are, sleeping rough, in the United States, there is no accurate picture of the number of homeless youths in the U.S. [1]. For most European countries this information is lacking as well. Although the estimation of homeless youths in the Netherlands varies widely, the minimum estimate of sheltered youths and street youths is 9000 [2]; this is 0,9% of the total population of youth between 18 and 23 years old [3].

Research has consistently shown that homeless youth, a highly vulnerable and heterogeneous population, suffer from a wide range of problems. Some youths are physically, emotionally and/or sexually abused, many have experienced family conflicts and have parents who were unwilling or unable to care for them [4,5]. Many youths have become dependent on services for homeless youth, have lived on the streets, or have found temporarily shelter with friends. The majority of homeless youths experience a low quality of life and lack the personal and social resources to hold their own successfully [6]. On average, they have little money to spend because of lack of income or high debts, a low level of education, are struggling to maintain a daily routine, and frequently, experience limited support of their social network which mostly consists of other homeless people [6-8]. Homeless youths report health problems, such as headaches and skin- and teeth problems [6]. Most studies found heightened rates of substance abuse [9]. The percentage of daily cannabis use among homeless youths in the Netherlands varies between 30% - 63% [8,10-12]. Also mental problems, such as depression, anxiety and psychological distress are common [9,13,14]. Many have lost trust in professionals and services [15]. Approximately 12-25% of homeless adolescents suffer from an intellectual disability [9,16].

Despite all the hardships homeless youths have suffered in their young lives and all the problems they are confronted with, some show remarkable resilience and many are able to make a successful transition into adulthood. Their own resources and personal power, and their ability to learn from their difficult experiences, are crucial factors in this success [17,18]. Both internal factors (e.g. self-esteem and self-efficacy, intelligence, perseverance) and external factors (e.g. affectional ties that encourage trust and autonomy) can contribute to the development of resilience [19]. Especially high self-esteem and self-efficacy are essential and seem to be acquired through supportive relationships [18]. Research on resilient children has shown that if a parent is incapable or unavailable to raise a child, other significant people can play an important role whether they are grandparents, siblings, care-providers or school teachers. In many situations it makes more sense to strengthen informal ties than to introduce additional layers of professionals [18-20].

To date, there are only few studies reporting on interventions among homeless youth: in a recent review on effective interventions only eleven published studies were found [21]. When looking at the interventions that are available for homeless youth in services for ambulant or residential care, the most promising are cognitive-behavioural interventions [21-24].

Yet, even though there is little information available on evidence-based interventions, some studies mention a supportive working relationship between professionals and youths as a crucial element in an effective intervention, [4,25-28]. Preliminary but promising results regarding higher levels of social connectedness and a trend towards decreased feelings of hopelessness were found in a study about the evaluation of the impact of a relationship-based intervention among homeless youth [29]. In the Netherlands in general, a variety of methods or approaches are being used in services for homeless youth, but there is no evidence for the effectiveness of these methods. Furthermore, research has shown that the available interventions hardly fulfill the needs of homeless youths [16]. When looking at the specific needs of homeless youths, they primarily wish to be taken seriously, to receive care from professionals who are committed, honest, authentic and flexible, and to have easy access to practical support and care [8,15,16,25].

In cooperation with professionals and homeless youths themselves, Wolf [30] developed a strengths based method, named: Houvast (Dutch for 'grip'). During the development of Houvast different activities were conducted to determine which elements are crucial for an intervention for homeless youth. Interviews were held with workers who homeless adolescents considered to be ‘effective’. In addition, focus groups with homeless youths were held as consultation of the target population is important in the process of developing an effective method [4]. Furthermore, a review [21] gave insight into available interventions and their scientific evidence.

Both scientific and practical evidence underlined the importance of a strengths based method. Houvast has been derived from a strengths based model that was originally developed for people with a psychiatric disability [31]. Currently, this model is being used with different subgroups of clients. Among abused women in Taiwan the strengths model contributes to a significant decrease in depression, a better life satisfaction and recovery from a sense of self [32]. Furthermore, a strengths based approach among persons with mental illness shows a positive association with number of hospitalizations, quality of life, social functioning and social support [33].

The main aim of Houvast is to improve the quality of life of homeless youths by focusing on their strengths and stimulating their capacity for autonomy and self-reliance. The fundamental assumption of the strengths perspective is that all people have strengths, talents and goals and that all environments consist of resources, people and opportunities. The strengths model emphasizes that the capacity for growth and recovery is an innate ability of human beings. Thus, even homeless youths who have experienced major live events can initiate change through exploring their inherent strengths and aspirations. The focus is on individual strengths rather than on problems and deficits. Young homeless people are their own director of the helping process and the working relationship is primary and essential for developing autonomy and self-reliance. The primary setting for work is the community which can be viewed as an oasis of recourses [30,34].

The present study aims to examine the effectiveness of Houvast in Dutch services for homeless youth and also to assess the fidelity of this strengths model. Studies that used fidelity scales have found better outcomes when services adhere closely to a model with specified critical components [35-37].

The research questions of the present study are:

1. Is Houvast more effective than care as usual in improving the quality of life among homeless youths?

2. Is Houvast more effective compared to care as usual regarding recovery in terms of mental and physical health, substance use, coping, resilience, psychological needs, care needs, working relationship with the professionals and attainment of personal goals?

3. To what degree is there consensus, between professionals and youths, on the degree of satisfaction with their working relationship?

4. What is the fidelity of the strengths based method and is there a positive association with the effectiveness of Houvast?

## Methods

### Study design

**Figure 1:**
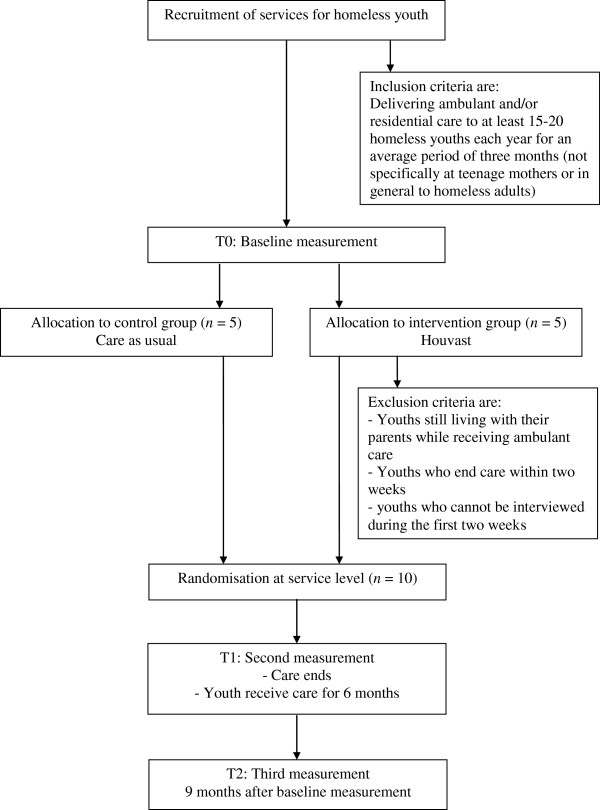
Flowchart of the randomization and measurement of the current study.

The effectiveness and fidelity of Houvast are investigated by means of a quasi-experimental research design with two groups and one baseline and two follow-up measurements (Figure 1). Both the intervention group and the control group (‘care as usual’) consist of Dutch services for homeless youth which deliver ambulatory and/or residential care. The services are randomly allocated to the intervention (*n* = 5) or control group (*n* = 5). Stratified randomization is used to ensure that services which deliver ambulant and/or residential care are equally distributed among each group. Randomization of homeless youths was not feasible since entire teams were trained prior to the baseline measurement in the strengths based method. Hence, a quasi-experimental research design is the best feasible design.

This study complies with the criteria for studies which have to be approved by an accredited Medical Review Ethics Committee region Arnhem-Nijmegen. Upon consultation, the Ethics Committee stated that due to the behavioural character of the intervention, the study was exempt from formal review (registration number 2011/260).

### Data collection

Measurements of primary and secondary outcomes are conducted among youths within the intervention group (*n* = 123) and the control group (*n* = 128) by means of three structured interviews. The first interview (T0) is conducted as soon as possible but within two weeks after the start of ambulant or residential care. Since the average duration of care delivered to homeless youths is three to six months, they are interviewed for a second time (T1) when services end care or when they have received care for a period of six months consecutively. The final follow-up interview (T2) takes place nine months after the first interview. In addition, professionals fill out an electronic questionnaire around T1 to obtain information about their perspective on the working relationship and the quality of life of a particular youth they provide care to. Based on the average number of homeless youths entering the participating services per year, the total period of the data collection is expected to be one year and nine months.

### Selection and inclusion criteria

Services for homeless youth in the Netherlands who were identified through a web search and through our network were all approached for participation in the study. Inclusion criteria for services to participate in the study were:

1) targeted at delivering ambulant and/or residential care to homeless youth (not specifically at teenage mothers or in general to homeless adults);

2) provision of care to at least 15–20 homeless youths per year;

3) provision of care for an average period of at least three months consecutively (thus excluding emergency shelters).

Of the 35 services that were approached, 18 services attended an introduction meeting about the study. Finally, ten services agreed to participate in the study and eight services declined the opportunity to participate. Reasons for non-participation of services were:

1) not able to generate the necessary financial contribution (the funder requires a financial contribution for services to participate in the study, e.g. to cover expenses for training);

2) already having implemented other working methods;

3) already participating in other studies;

4) pending reorganization within services or organizations.

All youths who enter the service and receive either ambulant or residential care from professionals are included in the study. Exclusion criteria are:

1) youths still living with their parents while receiving ambulant care;

2) youths who end care within two weeks;

3) youths who cannot be interviewed within the first two weeks after entering the service.

### Procedure

Participating services were informed about the study by an information brochure and two working visits of the researchers to the service. During the first working visit with the (team) managers, information about the study and the service was exchanged and a contact person at the facility for the study was established. During the second working visit, the researcher informed the entire team (team manager and professionals) about the study by way of a one-hour interactive presentation.

Homeless youths are approached for participation in the study according to the following procedure: professionals approach all youths entering the service by providing leaflets and drawing attention to a poster with information about the study. If a youth agrees to participate the contact person gives him or her more extended information about the study and sends contact information about this potential participant to the researcher who subsequently schedules an interview appointment. The contact person is familiar with the inclusion and exclusion criteria. To protect confidentiality of the provided data, a unique code is assigned to each participant.

Preferably, all interviews take place at the site of the service and, if this is not possible, at a public place such as a library. After giving written informed consent, all instruments including self-report measures, are administered by means of face-to-face interviews by trained research assistants who are unaware of randomization of the service to the intervention or control group and have experience or affinity with working with vulnerable people, including homeless youths. Youths receive ten euro for participating in the first interview, 20 euro for the second interview and 35 euro for the third interview.

After homeless youths leave the service, locating them for making a second and third interview appointment is challenging because they have often moved to different parts of the country. Therefore, permission is asked in the informed consent at baseline to collect different sources of contact information such as e-mail addresses, phone numbers (e.g. from friends and family) and social media accounts. A protocol for contacting the respondents is established for both follow-up measurements. It consists of the following steps: calling, sending a text message, calling again, calling the contact person of the service, calling again, sending an e-mail, using social media and/or calling friends or family. Youths are excluded if no interview appointment could be made after accomplishing these steps (after four weeks). Every two to four weeks the researcher calls the contact person to verify if participants have left the service (and could be approached for a second or third interview) and to check if their contact information is still up to date.

### Houvast

Youths in the experimental group receive care according to the Houvast method. Houvast aims at sustaining the recovery process of homeless youth by focusing on four conditional factors to achieve social quality: social inclusion, socio-economic security, social cohesion and social empowerment [30,38]. A trajectory comprises three parts, i.e., establishing a trusting working relationship and the setting of personal recovery goals based on a strength assessment, activities to support the personal recovery process of homeless youths and to achieve their goals, and evaluation and the ending or continuation of the trajectory. These parts are not free-standing but are interconnected, for instance, building a working relationship with youths and continuously updating the ‘strengths assessment’ and ‘recovery plan’ are important during the whole trajectory, as is constant reflection on activities and achievements. The strengths assessment and personal recovery plan are two primary tools in the strengths model that are meant to be executed in conjunction with each other. The strengths assessment allows youths and professionals to organize and make use of multiple strengths of youths in relation to resources in their living environment. After an estimation of the personal qualities of a particular youth is made, a personal recovery plan can help him or her to achieve their meaningful and important long term recovery goals step by step [30,31].

In this study several activities were carried out for the implementation of Houvast prior do the start of the data collection. Professionals in the experimental condition received a four day training provided by experienced trainers. Furthermore, two follow-up training days were organized for all trained professionals to further integrate the strengths based principles of Houvast and the fidelity criteria in their working methods, and to mutually exchange experiences about working with the Houvast intervention. In addition, internal coaches at the services within the intervention group were trained in getting familiar with the fundamental elements and competences of coaching to take responsibility for assuring and monitoring the implementation of Houvast and the model fidelity. Team leaders received a two and a half day training to learn what conditions need to be met within the organization in order to successfully implement Houvast. This is of particular importance because for a successful implementation of Houvast the organization and its management must adapt certain perspectives and practices. At the ‘pinnacle’ of the organization are the clients, and all organizational personnel are subservient to them. The managers are subservient to professionals. They need to be well grounded in the strengths based principles which are used by their professionals and to be able to integrate them in their policy [30,31].

### Care as usual

Youths in the control group receive care as usual. To get insight into care as usual, professionals fill in a questionnaire about education, years of working experience and the caseload. In addition, the contact person of each service in the control group answers general questions about the average intensity of care, coaching and the actual approach or method used.

### Measures among youths and professionals

#### Demographics

Several demographic characteristics are assessed among homeless youths, including age, gender, nationality, religion, ethnicity, marital status, family composition, residence permit and education. Demographics, such as gender, age, education and years of working experience are also assessed among professionals.

#### Quality of life

The primary outcome is quality of life, measured with the brief version of Lehman Quality of Life Interview [39-41]. This interview measures both objective and subjective quality of life on eight life domains: living situation, daily activities & functioning, family, social relations, finances, work & school, legal & safety issues and health. In addition, the interview contains a global measure of general quality of life which is asked at the beginning and at the end of the interview. The objective quality of life indicators can be categorized as two types: measures of functioning (frequency of social contacts or daily activities) and measures of access to resources and opportunities (income support of housing type). Youths are asked to indicate their subjective satisfaction of quality of life on a seven-point Likert scale, ranging from terrible (1) to delighted (7).

Professionals are asked to rate the quality of life of youth on the aforementioned seven-point Likert scale. For example: “What do you think of the quality of life of the client in general?” The psychometric properties are excellent and comparable to the full interview version [39,40,42].

#### Working relationship

To assess the working relationship between youths and professionals the Psychological Availability and Reliance on Adults (PARA) questionnaire [43] is administered. Both youths and professionals complete their own version of the PARA questionnaire to allow comparison of results. The PARA questionnaire consists of two subscales: perception of psychological availability and reliance. Youths have to answer on a 4-point Likert scale, varying from 1 (disagree) to 4 (agree). The PARA questionnaire was developed primarily for research among institutionalized adolescents [44]. The scale has been used among adolescent athletics as well, where the scale proved to be reliable [45].

### Measures among youths

#### Goals

To gain insight into the main goals homeless youths would like to attain, they are asked during the first interview to report three important personal goals for the next nine months. Next, for every goal a question regarding the importance of the goal and goal self-efficacy is administered which they can both answer on a 4-point Likert scale, varying from 1 (very unimportant or completely disagree) to 4 (very important or completely agree). Goal attainment is evaluated during the second and third interview by asking youths to what extent the earlier reported goals have been achieved. These questions are based on previous research on personal goals in relation to quality of life [46-48].

#### Changes in quality of life

During the second and third interview youths are asked about their possible changes in their quality of life since the last interview. The scale consists of twelve questions with a 7-point Likert scale, ranging from 1 (very much worsened) to 7 (very much improved). An example of a question is: “Has you quality of life been worsened, improved or unchanged compared to previous interview?” The life domains are based on the eight life domains of the Lehman quality of life questionnaire.

#### Substance use

The frequency and intensity of substance use is measured using the Dutch version of the European version of the Addiction Severity Index (EuropASI) [49]. Several questions regarding type of drugs have been added to the questionnaire. The EuropASI has frequently been used in surveys among homeless people with serious mental and/or addiction problems. Studies among substance-abusing populations show satisfactory results for the reliability and validity of the EuropASI [50].

#### Mental and physical health

The Brief Symptom Inventory (BSI-53) [51-53] is used to assess psychological distress. This instrument has widely been used in research among homeless youths and adults to measure mental health [23,54]. The BSI consists of 53 items, covering nine symptom dimensions: somatization, obsession-compulsion, interpersonal sensitivity, depression, anxiety, hostility, phobic anxiety, paranoid ideation and psychoticism. Items are measured on a 5-point Likert scale, ranging from 0 (not at all) to 4 (extremely). The internal consistency and the test-retest reliability are good and were established among a large population of psychiatric patients [53,55].

#### Intellectual disability

With the use of the Hayes Ability Screening Index (HASI) [56], a short screening of a possible intellectual disability of youths is obtained. The HASI, originally developed as a screening test to indicate possible intellectual disabilities among persons who come into contact with the criminal justice system, consists of three tests: 1) a spelling subtest; 2) a puzzle; and 3) a clock drawing test. In addition, the screening consists of four questions regarding already known learning difficulties. The HASI proves to be a valid and reliable screener [57-60].

#### Coping

The short version of the Cognitive Emotion Regulation Questionnaire (CERQ) [61] is administered to identify cognitive coping strategies after having experienced negative life events. The CERQ consists of nine different scales with each two items: self-blame, other-blame, rumination, catastrophizing, positive refocusing, planning, positive reappraisal, putting into perspective and acceptance. Items are measured on a 5-point Likert scale ranging from 1 (almost never) to 5 (almost always). The CERQ has widely been used in research among people with health problems [62]. The CERQ has been translated into other languages, among others Turkish [63] and Pursian [64]. The psychometric features were established among a large adult general population and the Cronbach’s alpha reliability coefficients for the subscales were acceptably high and the CERQ proves to be valid [61].

#### Psychological needs

The basic psychological needs scale [65] is used to measure the theoretical concept of self-determination. The scale consist of three subscales: competence, relatedness and autonomy. The total score reflects the extent to which participants are satisfied with the fulfillment of their basic psychological needs in general. Youths are asked to indicate their agreement with the 21 items on a 7-point Likert scale, ranging from 1 (not true at all) to 7 (definitely true). The scale has been used in many other studies [66]. Adequate factor structure, internal consistency, discriminant validity and predictive validity have been demonstrated among undergraduate students and Greek-speaking exercise participants [67,68]. The external validity of the questionnaire was ascertained by comparing scale scores with other with measures of well-being and worry [67].

#### Care needs

Care needs are assessed using a questionnaire developed by our research centre. The response categories were based on the format of the Short Form Quality of Life and Care questionnaire (QoLC) [69]. Needs are considered on the following 21 domains: housing, self care, finances, searching for work, daily activities, basic skills (reading, writing, calculating), household, transport, family contacts, social contacts, relationship with the children, help for own children, own safety, safety of other people, physical health, mental health, empowerment (assertiveness, self-defense courses), alcohol use, drug use, teeth and nutrition. For each domain, two questions were asked: 1) “Do you want help with . . . ?”, and 2) “Do you get help with . . . ?” The questionnaire has been used in research among homeless youths [6,70] and abused women [71].

#### Care use

Care use is assessed by using a questionnaire developed by our research centre. Youths are asked if they have used different types of care (e.g. psychological help, medical help, addiction treatment) at any time in their live and/or during the last six months and/or last 30 days.

#### Resilience

A positive secondary outcome, resilience, is measured with the Dutch Resilience scale (RS-NL) [72,73]. The Dutch scale consists of two subscales: 1) personal competence; and 2) acceptance of self and life. The 25-items are measured on a 4-point Likert scale, ranging from 1 (strongly disagree) to 4 (strongly agree). The Dutch version appears to be valid and reliable [73,74].

### Sample size and power calculation

To our knowledge no research among homeless youth has been carried out that used this quality of life instrument [39] to measure quality of life as the primary outcome measure. In comparable research among youths, in which a different instrument was used [75], an effect size of .95 was observed. Compared to baseline measurement, youths in the experimental group reported an increase in general life satisfaction of 6.45 (SD=5.89) at nine-month follow-up while controls reported a decrease of −2.25 (SD=11.61). Given that the setting and focus of the present study are different compared to Ferguson and Xie [75], a more conservative effect size was chosen. To calculate the required sample size, a mean improvement in the subjective quality of score of 0.74 (SD=1.48) is assumed. To detect this difference with 80% power (α = .05, two-sided), each group should comprise 63 participants. Considering potential loss of power due to clustering of data in services, power analyses revealed that at least 15–20 participants in each service (ICC = .05) are needed. Thus, taking a maximum of 30% attrition over time into account, 251 participants at baseline, and 178 participants at T1 are required.

### Model fidelity

To assess the model fidelity of the Houvast intervention, different assessments are conducted. During a one-day audit by two trained researchers to the service, the following activities are carried out: an interview with the team manager, an interview with the supervisor (in case a supervisor is established), a focus group with homeless youths, a file analysis, and an observation of a strengths based group supervision. Professionals, the supervisor and the team manager all fill out a questionnaire on the implementation of Houvast (e.g. questions about caseload, tasks and activities, use of strengths assessment and recovery plan) before the audit takes place.

Model fidelity is assessed with the fidelity scale of the strength based method. The fidelity scale consists of ten indicators: 1) case manager responsibilities; 2) caseload ratios; 3) supervisor to staff ratio and supervisor; 4) group supervision; 5) strengths assessment; 6) integration of strengths assessment; 7) personal recovery plan; 8) community contact; 9) naturally occurring resources; and 10) hope-inducing behaviours [31,35]. The scale consists of three subscales: structure, supervision/supervisor and clinical/service. Each indicator is rated on a 1 to 5 scale after gathering information from multiple sources. A total fidelity score is obtained by averaging the scores on the ten indicators. Each service receives an extensive report with specific recommendations on how to improve their model fidelity.

### Analyses

1) Differences in baseline characteristics (age, gender, education, nationality and ethnicity) between the intervention and control group will be checked by using Chi-square tests, Student’s t-tests and ANOVA's.

2) To examine if the Houvast method proves to be effective or not and to examine additional factors associated with quality of life of homeless youths, a two-level multilevel regression analysis will be used. Since data is clustered – youths are ‘nested’ in services – the analysis will be adjusted for the cluster effect at ‘service’ level. Analyses will be adjusted for potential confounders, such as gender, age, ethnicity, intellectual disability, duration of homelessness and model fidelity.

3) To assess the degree of consensus on the working relationship satisfaction between professionals and youths, a one-with-many design with multilevel analysis will be used. This design addresses the problems associated with non-independence and it accounts for interdependence between professionals and youths.

4) Regarding the fidelity assessment of the strengths based method of each service, all sources (e.g. questionnaires, interviews, observations) will be analysed and by averaging the scores on ten indicators a total score on a 5-point scale will be obtained, whereby a score of 4 reflects a sufficient model fidelity. The total score will also be used as a covariate when examining the effectiveness of Houvast.

## Discussion

There is a substantial need for an evidence-based method that is effective in addressing the specific needs of homeless youths [21]. The present study aims to fill this gap by examining the effectiveness and fidelity of the strengths based Houvast method.

This study possesses several strengths. First, the study is unique because it examines the effectiveness of a strengths based intervention among homeless youth. Second, this study will provide in-depth information about the development and recovery of homeless youths within a critical period of their lives. Third, the study also assesses the fidelity of the working method which has, to our knowledge, never been done before in intervention studies among homeless youth. A possible explanation for not finding effectiveness can be poor implementation of the Houvast method, resulting in low intervention fidelity. Therefore, in this study much attention is paid to implementation activities for both professionals and (team) managers. Furthermore, internal coaches at each service are established to supervise the implementation process. So far, the implementation activities have generated much enthusiasm and motivation among professionals, team managers and other stakeholders. Fourth, this study not only provides a framework for fidelity measurement but also results in a quality framework that can be used in other organizations for homeless youth. Fifth, the Houvast method could be improved based on the results of the effectiveness and fidelity measurements and will give organizations and also policy makers, for example in municipalities or ministry departments, handles for adjusting or changing their policies on homeless youths.

In addition, this study has also considerable methodological strengths as it is the first time that a larger sample (*n* = 251 at baseline) of homeless youths will be followed up for a period of nine months within an intervention and a control group. Youths will be interviewed by trained interviewers who are unaware of the randomization of services to the intervention of control group which minimizes potential interview bias.

Some limitations may be mentioned as well. First, due to the complex and unpredictable situation of homeless youth, it may be difficult to motivate them to participate in the follow-up interviews. Most youths leave the service within nine months and locating them to make an appointment for a final interview requires a large investment of researchers. By increasing the financial compensation for their time and effort to complete the interviews, they will hopefully remain motivated. Second, even though youths are interviewed for the first time during the first two weeks after entering the service, there is a small possibility that they were influenced in their scoring on the baseline measurement by whether they entered an experimental or a control service. We will inspect the baseline scores of both groups on significant differences in quality of life and the secondary outcomes, the last follow-up measurement will be conducted at nine months after baseline. Even though it is unique to follow homeless youths for this extensive period of time, a longer time span in which their recovery (or not) would have become more apparent would have been ideal. However, due to practical and financial constraints this was not feasible in the present study.

If the Houvast method proves to be effective in improving the quality of life of homeless youths, the study has strong practical relevance as the quality of work within services for homeless youth could be improved and more professionalized.

## Competing interests

The authors declare that they have no competing interests.

## Authors’ contribution

JW, SB and MK are responsible for the design of the study. MK is responsible for collecting data, performing statistical analysis and reporting of study results. She wrote the manuscript supervised by SB and JW who commented critically on the manuscript. All authors read and approved the final manuscript.

## Pre-publication history

The pre-publication history for this paper can be accessed here:

http://www.biomedcentral.com/1471-2458/13/359/prepub
